# Sequential Contribution of Parenchymal and Neural Stem Cell-Derived Oligodendrocyte Precursor Cells toward Remyelination

**DOI:** 10.3390/neuroglia1010008

**Published:** 2018-06-12

**Authors:** David R. Serwanski, Andrew L. Rasmussen, Christopher B. Brunquell, Scott S. Perkins, Akiko Nishiyama

**Affiliations:** 1 Department of Physiology and Neurobiology, University of Connecticut, 75 North Eagleville Road, Storrs, CT 06269-3156, USA; drsbeek@gmail.com (D.R.S.); andy.l.rasmussen@gmail.com (A.L.R.); christopherbrunquell@gmail.com (C.B.B.); scoperk11@gmail.com (S.S.P.); 2 Institute for Systems Genomics, University of Connecticut, Storrs, CT 06269, USA; 3 Institute for Brain and Cognitive Science, University of Connecticut, Storrs, CT 06269, USA

**Keywords:** demyelination, oligodendrocyte precursor, myelin, subventricular zone, NG2, neural stem cell

## Abstract

In the adult mammalian forebrain, oligodendrocyte precursor cells (OPCs), also known as NG2 glia are distributed ubiquitously throughout the gray and white matter. They remain proliferative and continuously generate myelinating oligodendrocytes throughout life. In response to a demyelinating insult, OPCs proliferate rapidly and differentiate into oligodendrocytes which contribute to myelin repair. In addition to OPCs, neural stem cells (NSCs) in the subventricular zone (SVZ) also contribute to remyelinating oligodendrocytes, particularly in demyelinated lesions in the vicinity of the SVZ, such as the corpus callosum. To determine the relative contribution of local OPCs and NSC-derived cells toward myelin repair, we performed genetic fate mapping of OPCs and NSCs and compared their ability to generate oligodendrocytes after acute demyelination in the corpus callosum created by local injection of α-lysophosphatidylcholine (LPC). We have found that local OPCs responded rapidly to acute demyelination, expanded in the lesion within seven days, and produced oligodendrocytes by two weeks after lesioning. By contrast, NSC-derived NG2 cells did not significantly increase in the lesion until four weeks after demyelination and generated fewer oligodendrocytes than parenchymal OPCs. These observations suggest that local OPCs could function as the primary responders to repair acutely demyelinated lesion, and that NSCs in the SVZ contribute to repopulating OPCs following their depletion due to oligodendrocyte differentiation.

## 1. Introduction

In the mammalian central nervous system (CNS), oligodendrocyte precursor cells (OPCs), also known as NG2 glia or polydendrocytes, represent a fourth major glial cell population that persists in the adult and continue to proliferate and generate myelinating oligodendrocytes throughout life, even after developmental myelination is largely completed (reviewed in [1,2]). Studies in the adult rat spinal cord have shown that OPCs proliferate in response to acute demyelination, and that the proliferated cells differentiate into oligodendrocytes [3–5]. Oligodendrocyte precursor cells in the telencephalon also contribute to remyelination of acute and chronic demyelinated lesions [3,6,7]. Genetic fate mapping has allowed more precise determination of the fate of OPCs under normal conditions [8–11] and established that the postnatal fate of OPCs is restricted to oligodendrocytes. This approach was also used to confirm that OPCs are an important source of remyelinating oligodendrocytes that repair demyelinated lesions [12,13].

In addition to local proliferating NG2 glia, the adult brain also contains neural stem cells (NSCs) that reside in the subventricular zone (SVZ). Neural stem cells in the adult SVZ pass through a transit-amplifying progenitor stage as they differentiate into neuroblasts that migrate through the rostral migratory stream (RMS) into the olfactory bulb, and their multipotential ability to generate astrocytes as well as neurons has been well characterized [14,15]. When demyelination occurs in the vicinity of the SVZ, cells in the SVZ become mobilized and may contribute to remyelination [16–18]. The availability of genetic fate mapping tools to identify the fate of OPCs and NSCs have allowed a direct comparison of the contribution of local OPCs and SVZ-derived cells toward myelin repair, and several studies were published recently with varying results. While two studies suggest a robust ability of NSCs to generate remyelinating oligodendrocytes [19,20], another recent study suggests that although SVZ cells proliferate in response to acute demyelination, they are unable to produce oligodendrocytes that contribute to myelin repair [21]. We have used Tg(Cspg4-creERTM) and Tg(Nes-creER^T2^) transgenic mice crossed to a reporter line and have compared the ability of local OPCs and nestin+ NSCs to generate oligodendrocytes in response to acute demyelination caused by α-lysophosphatidylcholine (LPC) injection into the adult corpus callosum. We show that local OPCs generate oligodendrocytes more rapidly than NSCs, whereas NSCs contribute to repopulating OPCs in the corpus callosum after myelin repair.

## 2. Materials and Methods

### 2.1. Animals

A bacterial artificial chromosome (BAC) transgenic mouse line expressing tamoxifen-inducible Cre in OPCs (Tg(Cspg4-creERTM) [9]; Jackson Laboratory stock #008538, Bar Harbor, ME, USA) was crossed to the cre reporter gt(ROSA)26Sor^tm1(EYFP)^ (YFP) [22] (Jackson Laboratory stock #006148) and maintained as double homozygotes. Tg(Nes-creER^T2^) mice were obtained from Dr. Amelia Eisch (University of Pennsylvania, Philadelphia, PA, USA) [23] and also crossed to YFP mice. Cre was induced by four consecutive days of intraperitoneal injection of 1 mg of 4-hydroxytamoxifen (4OHT, Sigma H-7904, St. Louis, MO, USA) every 12 h, as previously described [9].

To create acute demyelinating lesions, 1 μL of 2% LPC in phosphate-buffered saline (PBS) was stereotaxically injected into the right rostral corpus callosum using a Hamilton syringe at the coordinates (*−*1.3 mm from the bregma, 1 mm lateral, and 1.7 mm from the surface of the skull). Mice were sacrificed at 7, 14, and 28 days after LPC injection (7, 14, and 28 dpl) by intracardiac perfusion of 4% paraformaldehyde in 0.1 M sodium phosphate buffer, pH 7.4, containing 0.1 M L-lysine and 0.01 M sodium metaperiodate. Brains were isolated and post- fixed in the same fixative for 2 h at 4 C. For NG2creER;YFP mice, LPC injection was performed three days after the last 4OHT injection. For Tg(Nes-creER^T2^):gt(ROSA)26Sor^tm1(EYFP)^ mice, LPC injection was performed 28 days after 4OHT injection to allow for greater accumulation of YFP+ cells in the SVZ.

All animal procedures were approved by the Institutional Animal Care and Usage Committee in a protocol A16–018 “NG2 cells in the neural network” from June 24, 2016 through June 23, 2019.

### 2.2. Tissue Processing and Immunohistochemistry

Serial 50 μm coronal sections were cut on a vibratome (Leica VTS1000, Leica Biosystems Inc., Buffalo Grove, IL, USA) and stored at *−*20 °C in 98-well plates in cryostorage solution consisting of 10 g of polyvinylpyrolidone, 500 mL 0.2 M sodium phosphate buffer, pH 7.4, 300 g sucrose, and 300 mL ethylene glycol in 1 L. Sections were processed for immunohistochemistry as previously described [9]. The primary antibodies used are listed in Table 1. Secondary antibodies were Alexa 488-labeled anti-chick antibody (1:1000), Cy3-labeled anti-mouse, rabbit, or goat (1:200), and Cy5- (1:100) or Alexa 647-labeled (1:200) anti-mouse, rabbit, or goat antibodies from Jackson ImmunoResearch (West Grove, PA, USA). Five or six sections from each mouse that were 600 μm apart were labeled with the antibodies and mounted with Vectashield containing 4’,6-diamidino-2-phenylindole dihydrochloride (DAPI, Vector Labs, Burlingame, CA, USA). Stained sections were analyzed on Leica TCS SP2 or SP8 confocal microscope and Zeiss Axiovert 200 M with ORCA ER camera and apotome (Carl Zeiss Microscopy, Jena, Germany).

### 2.3. Cell Quantification

Demyelinated lesions were assessed by a combination of immunolabeling for myelin basic protein (MBP) and non-phosphorylated neurofilaments. For quantification, a series of tiled confocal *z*-stack images were collected over a *z*-distance of 20–30 μm, encompassing the lesion from at least two sections for each animal. Cell numbers were estimated by scoring each YFP+ cell for the expression of specific antigens within a defined area for each section. The area for quantification was defined as the area that lacked MBP immunofluorescence in adjacent sections for the early time points. For later time points, the lesioned and repaired areas were defined as areas that contained dense reactive NG2 glia and astrocytes. To obtain the density of the cells, the area from which cell numbers were obtained was calculated using ImageJ [24] and multiplied by the thickness of the *z*-stack.

### 2.4. Statistical Analysis

Quantification results are expressed as mean *±* standard deviation. Statistical analyses were performed using two-way analysis of variance (ANOVA) with uncorrected Fisher’s least significant difference (LSD) test for the quantification of % YFP+ cells that were NG2+ or CC1+ and the density of YFP+ NG2+ and YFP+ CC1+ cells. Student’s t-test (two-way, unpaired) was used for the quantification of the percentage of CC1+ cells derived from NG2+ or nestin+ precursor cells over 14 days. Sample sizes ranged from three to four.

## 3. Results

### 3.1. Evolution of LPC-Induced Demyelinated Lesion

**Figure 1.**
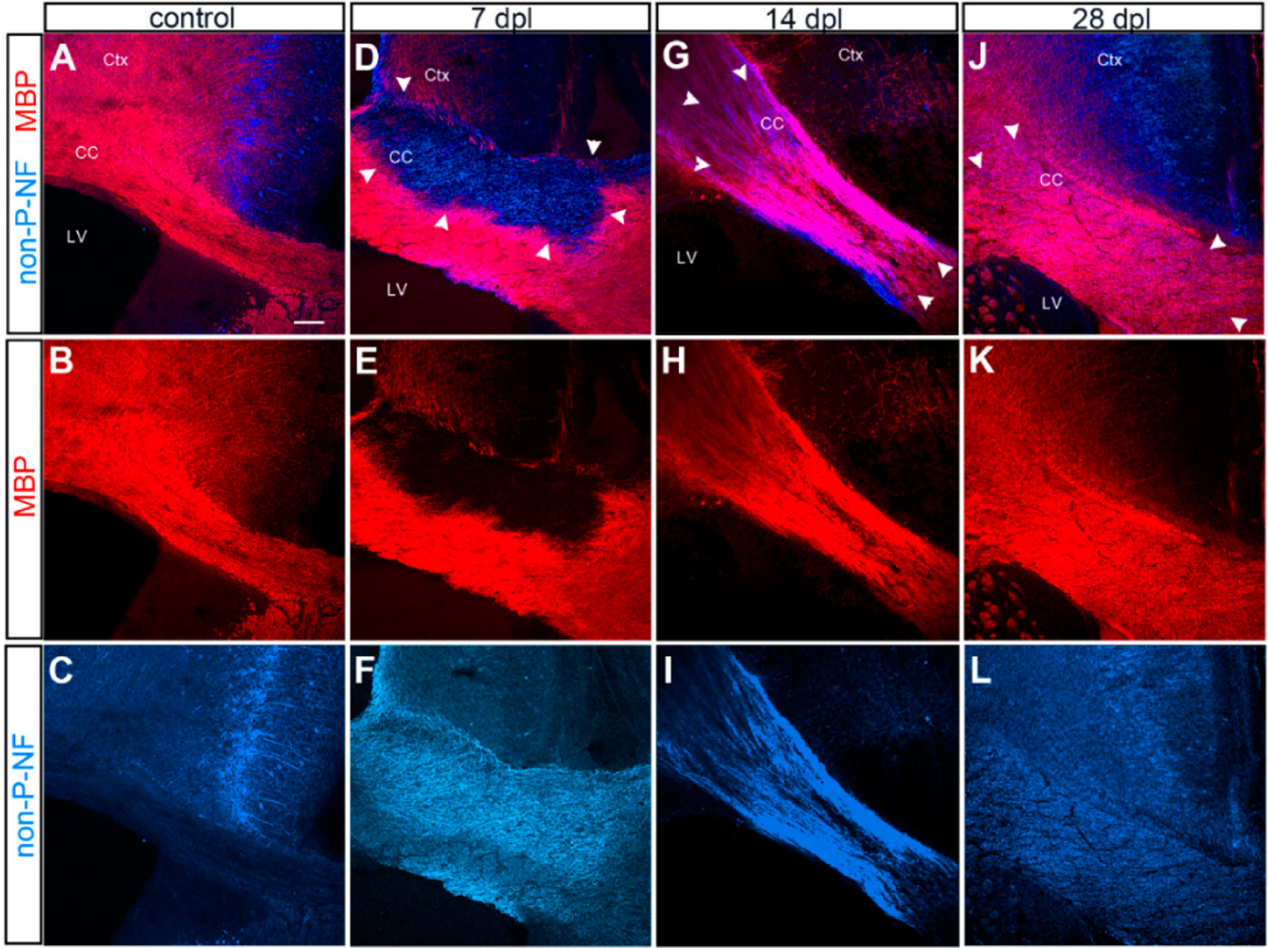
Evolution of α-lysophosphatidylcholine (LPC)-induced demyelinated lesion. Immunofluorescence labeling for myelin basic protein (MBP) and non-phosphorylated neurofilaments. (**A**–**C**) Control unlesioned brain. Intact MBP+ myelin in the corpus callosum. Non-phosphorylated neurofilaments are restricted to the neurons in the cingulate cortex. Ctx: cortex, CC: corpus callosum, LV: lateral ventricle. (**D**–**F**) Demyelinated corpus callosum at 7 days post lesioning (dpl) showing a well-defined lesion lacking MBP and upregulated non-phosphorylated neurofilaments. Boundary of the lesion is indicated by arrowheads. (**G**–**I**) Demyelinated corpus callosum at 14 dpl showing partial remyelination, characterized by uneven MBP labeling and persistent presence non-phosphorylated neurofilaments. (**J**–**L**) Remyelinated corpus callosum at 28 dpl showing uniform MBP labeling and reduced levels of non-phosphorylated neurofilaments, though they are higher than unlesioned corpus callosum. Scale bar: 100 μm.

In the normal adult mouse corpus callosum, MBP was robustly detected in the corpus callosum, and there was little detectable non-phosphorylated neurofilaments, with the exception of axons in the cingulate cortex (Figure 1A–C). Injection of LPC into the corpus callosum resulted in focal demyelination, characterized by a well demarcated loss of MBP immunoreactivity at 7 days after lesioning (dpl) (Figure 1D–F, arrowheads in D), accompanied by increased immunoreactivity for non-phosphorylated neurofilaments, which have been shown to increase in demyelinated axons [25]. By 14 dpl, the area of demyelinated lesion had decreased, and a substantial amount of myelin had been regenerated, while non-phosphorylated neurofilaments were still present. By 28 dpl, the lesion was indistinguishable from the surrounding myelinated region in the majority of the animals. The evolution of the lesion was consistent with previously published reports (for example, [26]).

### 3.2. Contribution of Local OPCs to Remyelinating Oligodendrocytes

**Figure 2.**
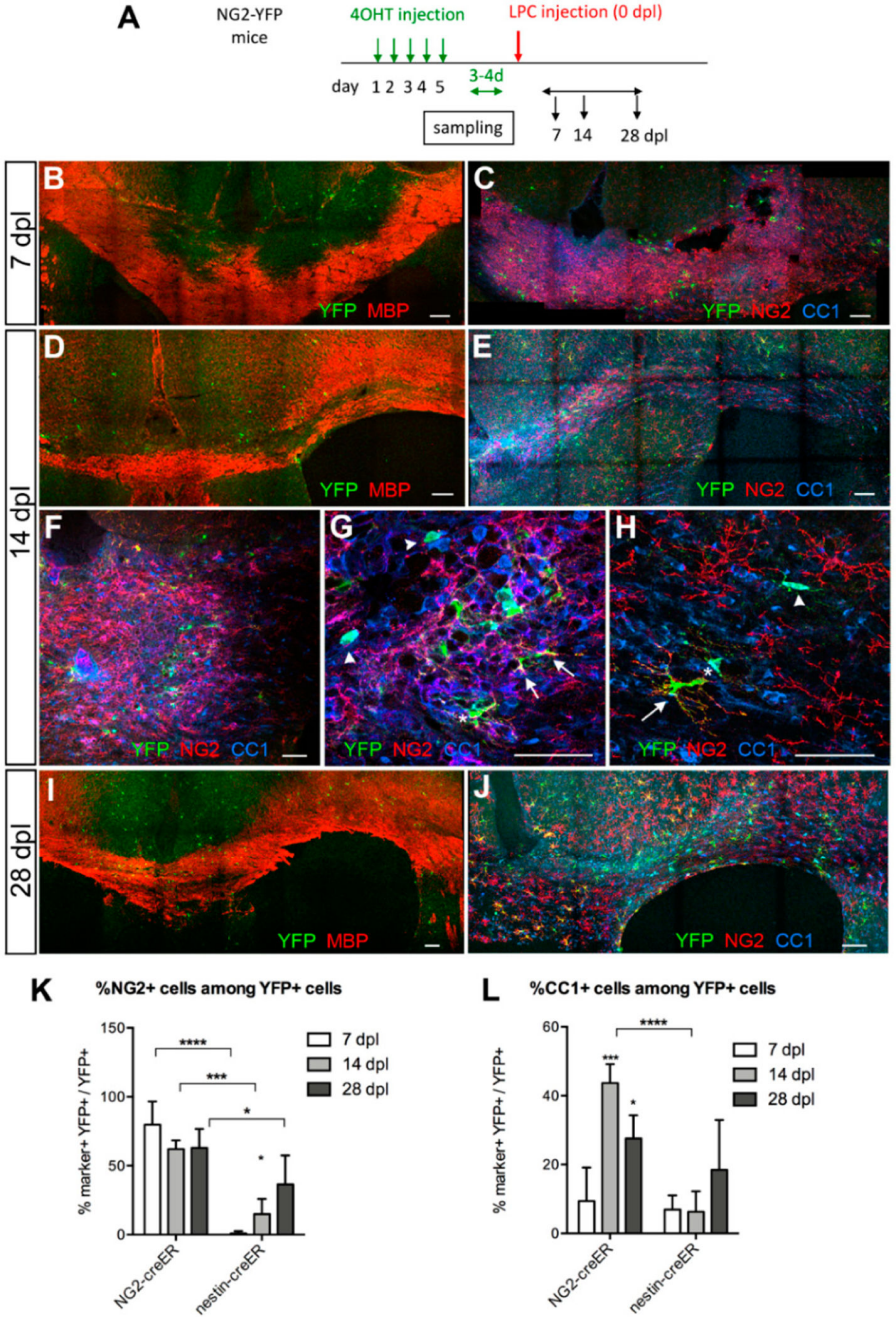
Response of local oligodendrocyte precursor cells (OPCs) to LPC-induced demyelination in the corpus callosum. (**A**) Scheme showing the experimental outline. (**B**,**C**) Lesion at 7 dpl. Low magnification images of immunolabeling for MBP and yellow fluorescent protein (YFP) showing an area of demyelination (**B**) and immunolabeling for YFP, NG2, and CC1 showing scattered YFP+NG2+ cells in the lesion (**C**). (**D**–**H**) Lesion at 14 dpl. Low magnification images of immunolabeling for MBP and YFP showing partially remyelinated lesion (**D**), characterized by uneven MBP staining, and immunolabeling for YFP, NG2, and CC1 showing increased number of YFP+ cells in the lesion (**E,F**). Higher magnification shows a significant proportion of YFP+ cells express CC1 (arrowheads), while other YFP+ cells are NG2+ (arrows). Some YFP+ cells express both NG2 and CC1 (asterisks) at varying ratios. (**G**) is a higher magnification of the lesion. (**H**) is from a site further away from the lesion. (**I**,**J**) Lesion at 28 dpl. Immunolabeling for MBP and YFP shows largely repaired lesion (**I**) and a cluster of YFP+CC1+ cells in the center of the repaired lesion while YFP+ NG2+ cells are seen at the periphery (**J**). Scale bars: 100 μm for (**B**–**F**) and (**I**,**J**); 50 μm for (**F**–**H**). (**K**,**L**) The proportion of YFP+ cells that were NG2+ (**K**) or CC1+ (**L**) in Tg(Cspg4-creERTM;gt(ROSA)26Sor^tm1(EYFP)^ (NG2-YFP) and Tg(Nes-creER^T2^);gt(ROSA)26Sor^tm1(EYFP)^ (nestin-YFP) mice. * *p* < 0.05, *** *p* < 0.001, **** *p* < 0.0001. *n* = 3, two-way ANOVA, uncorrected Fisher’s least significant difference (LSD) test.

To investigate the extent to which local OPCs contribute to remyelination, we used Tg(Cspg4-creERTM;gt(ROSA)26Sortm1(EYFP) (NG2-YFP) double transgenic mice. The fate of local OPCs was followed during the course of demyelination and remyelination by activating cre-mediated recombination and YFP expression in OPCs 3–4 days prior to LPC injection (Figure 2A). One day after the last tamoxifen injection, 40–50% of OPCs in the corpus callosum were YFP+ [9]. We induced cre before LPC injection to avoid activating YFP expression in macrophages that could also express NG2 [27]. This regimen also minimized labeling of SVZ progenitor cells that were mobilized and upregulated NG2 expression after demyelination.

To determine the dynamics of oligodendrocyte differentiation from parenchymal OPCs, we determined the percentage of YFP+ cells that were NG2+ oligodendrocyte precursor cells or CC1+ differentiated oligodendrocytes at 7, 14, and 28 dpl. At 7 dpl, when there was a well-defined demyelinated lesion characterized by a lack of MBP reactivity, clusters of YFP+ cells were seen in and around the lesion suggestive of local proliferation (Figure 2B,C). Among the YFP+ cells, 80% were NG2+, while only 10% were CC1+ oligodendrocytes (Figure 2E,K,L). By 14 dpl, the proportion of YFP+ cells that were NG2+ decreased to 62%, while the proportion of YFP+ cells that were CC1+ increased to 44% (Figure 2D,E,K,L), and clusters of YFP+ CC1+ cells were found inside the lesion (Figure 2F,G). Some YFP+ cells expressed both NG2 and CC1, which suggests that OPCs were rapidly differentiating into CC1+ oligodendrocytes. Outside the lesion, both YFP+ NG2+ cells and YFP+ CC1+ oligodendrocytes were detected as well (Figure 2H). The proportion of YFP+ cells that were CC1+ in and around the lesion at 14 dpl was more than 4-fold greater than that at 7 dpl, suggesting that OPCs that were recruited to the lesion were undergoing oligodendrocyte differentiation. At 28 dpl, the proportion of YFP+ cells that were NG2+ remained at 63%, while the proportion of YFP+ cells that were CC1+ decreased slightly to 30% (Figure 2I–L).

**Figure 3.**
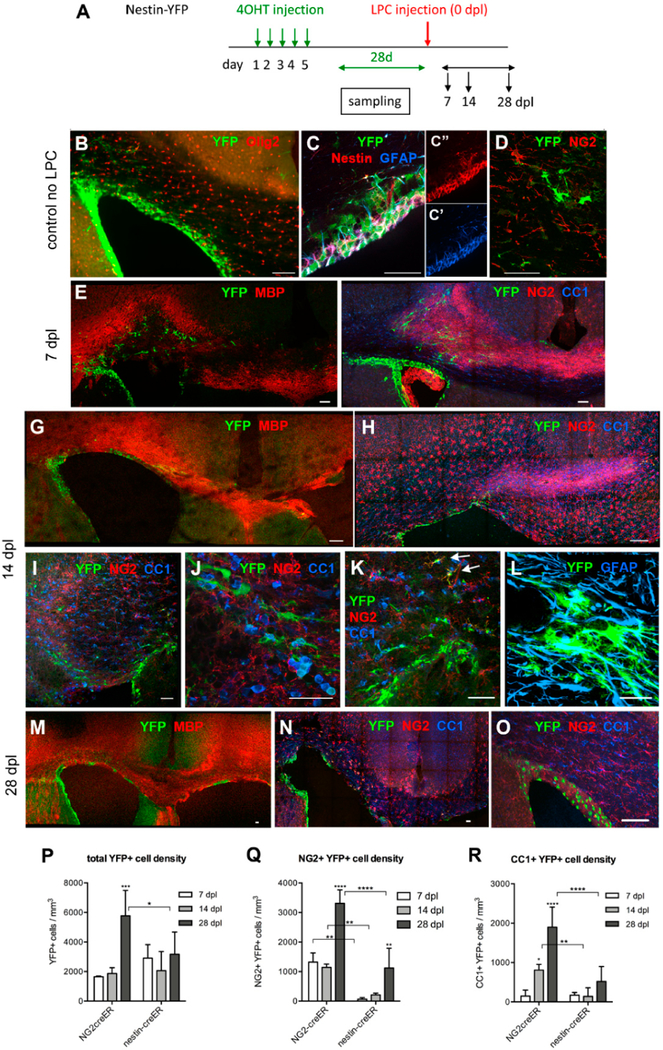
Response of subventricular zone (SVZ) cells to LPC-induced demyelination in the corpus callosum. (**A**) Scheme showing the experimental outline. (**B**–**D**) The distribution and phenotype of YFP+ cells prior to LPC injection. The majority of the YFP+ cells are found in the SVZ (**B**) and very few of the YFP+ cells expressed Olig2. The majority of the YFP+ cells expressed nestin and GFAP (**C**) but not NG2 (**D**). (**E**,**F**) Lesion at 7 dpl. Low magnification images of MBP and YFP immunolabeling showing an area of demyelination with YFP+ cells mostly above the lateral ventricle (**E**) and immunolabeling for YFP, NG2, and CC1 showing that most of the YFP+ cells are neither NG2+ nor CC1+. There is strongly upregulated NG2 immunoreactivity throughout the lesion. Note that YFP+ cells line the SVZ. Some YFP+ cells appear to be migrating toward the needle track (arrows). (**G**–**L**) Lesion at 14 dpl. Low magnification of immunolabeling for MBP and YFP showing partially remyelinated lesion (**G**) and immunolabeling for YFP, NG2, and CC1 showing a slightly increased number of YFP+ cells in the lesion (**H**). Higher magnification shows that most of the YFP+ cells were confined to the lesion border (**I**). Higher magnification of **I** shows that most of the YFP+ cells did not express NG2 or CC1. (**J**) A region from the periphery of the lesion above the lateral angle of SVZ showing two YFP+ NG2+ cells (arrowheads). Some of the large, YFP+ cells were glial fibrillary acidic protein (GFAP)+ (**L**). (**M**–**O**) Lesion at 28 dpl. YFP and MBP labeling show remyelinated lesion with increased YFP+ cells in the lesion (**M**) and that the majority of the YFP+ cells were NG2+ (**N**). (**O**) Control mouse injected with PBS and stained for YFP, NG2, and CC1 at 28 dpl, showing that the majority of the YFP+ cells are confined to the SVZ and few YFP+ cells were detectable in the corpus callosum. Scale bar: 50 μm. (**P**–**R**) The density of YFP+ cells (**P**), YFP+ NG2+ cells (Q), and YFP+ CC1+ cells at 7, 14, and 28 dpl in NG2-YFP and nestin-YFP mice. * *p* < 0.05, ** *p* < 0.01, *** *p* < 0.001, **** *p* < 0.0001. *n* = 3–4, two-way ANOVA, uncorrected Fisher’s LSD.

When we examined the density of total YFP+ cells and YFP+ NG2+ cells, there was no significant difference between 7 and 14 dpl, but a 3-fold increase at 28 dpl (Figures 2J and 3P,Q). By contrast, the density of YFP+ CC1+ cells continued to rise from 7 to 28 dpl (Figure 3R). This suggests that after demyelination local OPCs already proliferated by 7 dpl and actively generated oligodendrocytes over the course of four weeks. The increase in the density of YFP+ NG2+ cells at 28 dpl likely reflects continued proliferation of local OPCs that existed prior to demyelination, as well as migration of OPCs from the surrounding areas into the lesion and the surrounding corpus callosum.

### 3.3. Contribution of SVZ-Derived Cells to Remyelinating Oligodendrocytes

#### Distribution and Phenotype of YFP+ Cells in Tg(Nes-creER^T2^);gt(ROSA)26Sor^tm1(EYFP)^ Mice

To assess the contribution of SVZ progenitor cells to remyelination, we performed a similar experiment in Tg(Nes-creER^T2^);gt(ROSA)26Sor^tm1(EYFP)^ (nestin-YFP) mice. We induced YFP in nestin+ progenitor cells in the SVZ 28 days prior to LPC injection, as the YFP labeling efficiency was too low immediately after tamoxifen injection (Figure 3A). At 28 days after tamoxifen injection, YFP expression was detected in 30–50% of nestin+ cells in dorsal and dorsolateral SVZ, as well as in medial and latera SVZ and the rostral migratory stream (RMS). Thus, the YFP labeling efficiency among nestin+ NSCs with this induction protocol was comparable to the efficiency among OPCs in NG2cre-YFP mice. In this study, we focused on YFP+ cells in the dorsal and dorsolateral SVZ and the corpus callosum of nestin-YFP mice. Almost 90% of the YFP+ cells resided in the SVZ, where they expressed nestin and glial fibrillary acidic protein (GFAP), two proteins known to be expressed by NSCs in the SVZ (Doetsch et al., 1999) (Figure 3B,C). A small fraction of YFP+ cells in the SVZ expressed the oligodendrocyte transcription factor Olig2, and most of the Olig2+ YFP+ cells were located in the outer layers of the SVZ. Of the YFP+ cells, 11.7% were in the corpus callosum, mostly above the dorsolateral angle of the SVZ (Figure 3B, upper left). Among the YFP+ cells in the corpus callosum, 64% were GFAP+, 47% were nestin+, and 77% were positive for the polysialylated form of neural cell adhesion molecule (PSA-NCAM), which is known to be expressed by neuroblasts and neural progenitor cells in the neurogenic niches of the SVZ and dentate subgranular zone [28,29], as well as on growing axons that are defasciculating at the target [30,31]. Among the YFP+ cells in the corpus callosum, 7.5% expressed NG2 (Figure 3D), and only 0.8% of the total YFP+ cells in the corpus callosum and in the dorsal and dorsolateral SVZ expressed NG2 prior to LPC injection.

### 3.4. Response of Nestin+ SVZ Cells to Acute Demyelination

To examine whether nestin+ SVZ progenitor cells generated OPCs that were recruited to acutely demyelinated lesions in the corpus callosum, we examined the changes in YFP+ NG2+ and YFP+ CC1+ cells in nestin-YFP mice over the course of four weeks after LPC injection. At the time of peak demyelination at 7 dpl, we saw an infiltration of YFP+ cells in the demyelinated corpus callosum (Figure 3E). In addition to scattered YFP+ cells in the demyelinated corpus callosum, there was a line of YFP+ cells that extended into the injection site in the more rostral sections that had the needle track (e.g., Figure 3F, arrowheads). Fewer than 3% of the YFP+ cells were NG2+ (Figures 2K and 3F), in stark contrast to NG2-YFP mice in which 80% of the YFP+ cells were NG2+ (Figure 2K).

By 14 dpl, 18% of the YFP+ cells had become NG2+, but the density of YFP+ NG2+ cells remained less than one-fourth of that in NG2-YFP mice (Figure 3G,H,Q). Many of the YFP+ cells, including those found in the lesion, had large polygonal cell bodies that were morphologically distinct from oligodendrocyte lineage cells, and some expressed GFAP (Figure 3J–L). Some YFP+ NG2+ cells with typical polydendrocyte morphology were found at the border of the lesion (Figure 3K, upper right). The percentage of YFP+ CC1+ oligodendrocytes in nestin-YFP mice did not increase from 7 dpl to 14 dpl (Figure 2L). Neither did the density of YFP+ CC1+ cells increase from 7 to 14 dpl (Figure 3R) and remained 5.7-fold lower than that in NG2-YFP mice.

At 28 dpl, the most notable change was that the density of YFP+ NG2+ cells in nestin-YFP mice increased 5.3-fold over that at 14 dpl (Figure 3Q). The average density of YFP+ CC1+ oligodendrocytes in these mice at 28 dpl was three-fold higher than at 14 dpl but did not reach statistical significance (*p* = 0.0876) and remained 3.6-fold lower than the density of YFP+ CC1+ cells in NG2-YFP mice at 28 dpl. Thus, between 14 and 28 dpl, NSC-derived cells seemed to be most actively generating OPCs, while the parenchymal OPCs were actively producing oligodendrocytes between 7 and 14 dpl, having expanded the precursor population earlier, by 7 dpl. The density of NSC-derived OPCs in the lesion at 28 dpl reached a comparable level to that of OPCs derived from the local population seen at 7 and 14 dpl. These observations indicate that after acute demyelination, local parenchymal OPCs initially responded more rapidly and robustly than SVZ-derived cells to produce oligodendrocytes, and the response of NSCs lagged behind by 2–3 weeks.

To further evaluate the contribution of parenchymal OPCs and NSC-derived cells in the supply of oligodendrocytes to the lesion, we estimated the proportion of total CC1+ oligodendrocytes in the dorsal aspect of the corpus callosum that were generated from parenchymal OPCs or NSCs at 14 dpl, as previously estimated in the normal adult corpus callosum [9,11]. We obtained the percentage of CC1+ oligodendrocytes that were YFP+ in NG2-YFP and nestin-YFP mice at 14 dpl and adjusted for the recombination efficiency in the respective mice. We found that 0.222% and 0.060% of CC1+ oligodendrocytes were generated from local OPCs and nestin+ NSCs, respectively, during the two weeks after demyelination. Thus, NG2-YFP mice had generated 4.3 times more CC1+ oligodendrocytes than nestin-YFP mice (*p* = 0.0188). This corroborates the other quantifications and indicates that after acute demyelination, local OPCs were more likely to contribute to remyelinating oligodendrocytes than SVZ-derived oligodendrocyte lineage cells.

The density of total YFP+ cells and YFP+ NG2+ cells in nestin-YFP mice was more than two-fold greater at 28 dpl than at 14 dpl. In contrast to clusters of YFP+ cells found at 7 and 14 dpl, many YFP+ NG2+ cells at 28 dpl were distributed more uniformly throughout the remyelinated and neighboring corpus callosum (Figure 3M,N). In PBS-injected mice, very few YFP+ cells were detected in the corpus callosum, similar to uninjured mice prior to LPC injection (Figure 3O). This suggests that demyelination triggered SVZ cells to generate oligodendrocyte lineage cells, and that the SVZ-derived cells were likely to be contributing to the replenishment of the OPC population that had been depleted due to their differentiation into remyelinating oligodendrocytes.

### 3.5. Transient Migration of NSC-Derived Neuroblasts into Corpus Callosum after Demyelination

**Figure 4.**
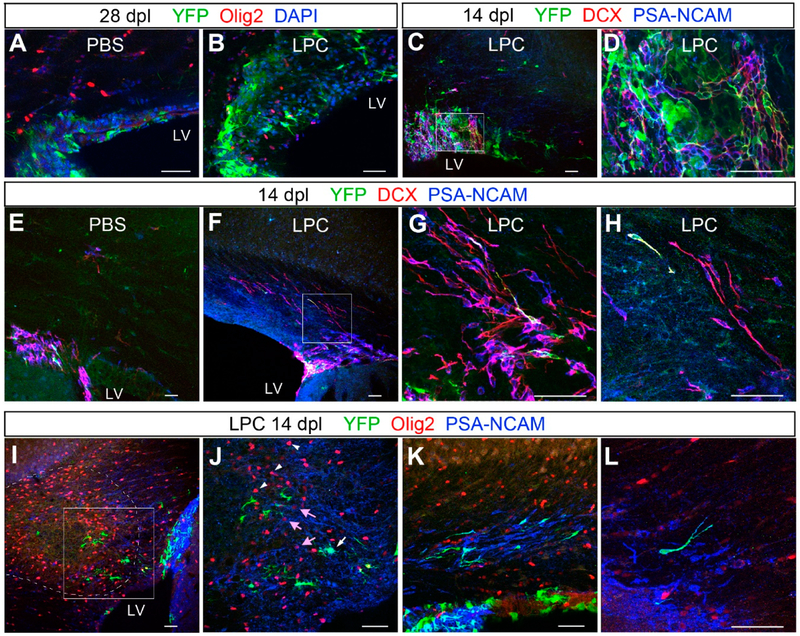
Changes in subventricular zone (SVZ) cells after LPC-induced demyelination. (**A**,**B**) Immunolabeling for YFP, Olig2, and DAPI showing increased thickness of the dorsal SVZ 28 days after LPC injection (**B**) compared with PBS-injected control (**A**). Very few Olig2+ cells are found in the control SVZ after PBS injection, whereas several Olig2+ cells are detected in the thickened SVZ after LPC injection. (**C**,**D**) Immunolabeling for doublecortin (Dcx) and PSA-NCAM showing that many of the YFP+ cells co-express Dcx and PSA-NCAM. (**D**) represents a higher magnification of the boxed area in **C**. (**E**–**H**) Immunolabeling for YFP, Dcx, and (PSA-NCAM) showing that most of the Dcx/PSA-NCAM+ cells are confined to the SVZ in control (**E**), whereas LPC-injected animals show a greater number of Dcx+ PSA-NCAM+ cells migrating dorsally into the SVZ (**F**–**H**). (**H**) is a higher magnification image of the boxed area in (**F**). (**I**,**J**) Immunolabeling for YFP, Olig2, and PSA-NCAM near the lesion (dotted line) at 14 dpl. There is a dense cluster of PSA-NCAM+ cells in the lateral angle of the SVZ (right side in I). Higher magnification of the boxed area in (**I**), showing a YFP+ cell that is also PSA-NCAM+ (**J**, white arrow). There is a cluster of strongly Olig2+ cells inside the lesion (arrowheads in (**J**)), and weakly Olig2+ PSA-NCAM+ cells are detected at the lesion border (pink arrows in (**J**)). (**K**,**L**) PSA-NCAM+ cells, some of which are YFP+, appear to be migrating in the corpus callosum in clusters (**K**), and many of them have a tadpole-shaped unipolar morphology (**L**). Scale bar: 50 μm.

We noticed that the dorsal SVZ was thicker in LPC-injected mice than in control PBS-injected mice. When we measured the height of the dorsal SVZ at 28 dpl, it was approximately two-fold greater in LPC-injected mice compared to PBS-injected control mice (Figure 4A,B), suggesting that there was sustained proliferative activity in the SVZ after remyelination had occurred. We examined the cellular composition in the dorsal SVZ that was expanded after demyelination. Previous studies had described PSA-NCAM+ and doublecortin (Dcx)+cells that emerged from the SVZ into acutely demyelinated corpus callosum [16,18,32]. In the SVZ at 14 dpl, we also found large clusters of PSA-NCAM+ and Dcx+ cells, many of which co-expressed the two antigens and had small, round, or oval cell bodies and long slender processes (Figure 4C,D). Some of them were also YFP+ in nestin-YFP mice. These YFP+ cells had a distinct morphology from flat polygonal NSCs that were YFP+ nestin+, suggesting that they were progeny of the NSCs.

In the corpus callosum of control PBS-injected mice, there were very few PSA-NCAM+ Dcx+ cells at 14 dpl (Figure 4E). In LPC-injected mice, a larger number of PSA-NCAM+ Dcx+ cells were seen migrating into the corpus callosum, mostly from the dorsolateral angle of the SVZ (Figure 4F–H). Their processes were oriented parallel to the axons in the corpus callosum, and many had the typical unipolar tadpole shape, as previously described [16,18], consistent with their neuroblast identity (see Figure 4H, top left cell that is also YFP+).

To examine whether PSA-NCAM+ or Dcx+ cells contributed to the repair of the demyelinated lesion, we examined the phenotype and distribution of YFP+ PSA-NCAM+ cells in nestin-YFP mice after demyelination. At 14 dpl, many PSA-NCAM+ cells were found at the periphery of the lesion, while very few were seen in the core of the lesion where there was a high density of Olig2+ cells (Figure 4I,J). While the majority of the cells inside the lesion that were strongly positive for Olig2 did not express PSA-NCAM (Figure 4J, arrowheads), a few weakly Olig2+ cells around the lesion also expressed PSA-NCAM (Figure 4J, arrows). Most of the PSA-NCAM+ cells around the lesion were YFP-negative in nestin-YFP mice, suggesting that they had been generated from cells that were already neuroblasts or transit amplifying cells by the time of LPC injection. There were occasional clusters of PSA-NCAM+ cells oriented parallel to each other but not necessarily in the direction of the demyelinated lesion (Figure 4K,L). Some of the PSA-NCAM+ cells had the typical tadpole morphology, with their leading edge pointing away from the SVZ, suggestive of emigration from the SVZ into the corpus callosum. At 28 dpl, fewer than 2% of the PSA-NCAM+ cells expressed Olig2, and the majority of PSA-NCAM+ cells appeared to remain as migrating neuroblasts. These findings suggest that acute demyelination triggered emigration of PSA-NCAM+ Dcx+ neuroblasts from the SVZ. However, during the four weeks after demyelination, we did not detect a significant contribution of YFP+ PSA-NCAM+ cells into the lesion, even at 28 dpl when we saw a significant number of YFP+ NG2+ cells in the corpus callosum. This makes it more likely that many of the NSC-derived OPCs that populated the corpus callosum after remyelination had directly differentiated into oligodendrocyte lineage cells from NSCs, rather than having passed through an intermediate stage of PSA-NCAM+ Dcx+ neuroblasts.

## 4. Discussion

We have shown that after chemically induced acute demyelination in the adult corpus callosum, local OPCs were rapidly recruited to the demyelinated lesion where they generated oligodendrocytes by 14 dpl. By contrast, nestin+ SVZ cells expanded and migrated into the lesion but did so after a delay of two weeks and continued to generate OPCs in the corpus callosum through 28 dpl when remyelination had already occurred. These observations suggest that local OPCs provide the major source of myelin repair after acute demyelination, while NSCs in the SVZ are an important source of repopulating the OPC population following their loss due to their differentiation into remyelinating oligodendrocytes.

### 4.1. Oligodendroglial Fate of Local Parenchymal OPCs and NSCs in the SVZ

It is well established that parenchymal OPCs that reside in the corpus callosum proliferate and generate remyelinating cells after an acute demyelinating injury created by local LPC injection [6], reviewed in [1,33]. There is also evidence that PSA-NCAM+ and/or Dcx+ neuroblasts in the SVZ migrate toward an acutely demyelinated lesion in the corpus callosum, although it has remained uncertain as to whether they generate remyelinating cells [16,18,34], see below).

The cuprizone-induced demyelinating model is another commonly used demyelination model with a more protracted time course. In mice fed on a cuprizone diet, demyelination occurs over five to six weeks, and remyelination ensues over the two to six weeks after the mice are returned to normal diet [35,36]. Moreover, if mice are continued on the cuprizone diet for 12 weeks, persistent demyelination occurs with poor remyelination [7]. Two recent studies used Tg(Nes-creERT2) mice and Tg(Pdgfra-creERT2) mice crossed to the ROSA-YFP reporter mice to compare the fate of NSCs and parenchymal OPCs, respectively, during the remyelination phase of cuprizone-induced demyelination [19,20]. In both studies, the progeny of NSCs, particularly those in rostral corpus callosum, robustly generated remyelinating oligodendrocytes to a greater extent than local OPCs. Furthermore, in regions above the SVZ, NSC-derived oligodendrocytes produced thicker myelin than local NG2 cell-derived oligodendrocytes [19].

On the contrary, another recent study used Tg(GFAP-creERT2) crossed to the YFP reporter to examine the fate of GFAP+ NSCs in the SVZ following LPC-induced acute demyelination [21]. Despite a robust proliferative response in the SVZ in both the young and old mice, NSC-derived cells generated only a very small fraction of oligodendrocyte lineage cells, and the vast majority of oligodendrocyte lineage cells in the remyelinated lesion were generated from parenchymal cells [21], consistent with an earlier imaging study [37]. In our LPC-induced demyelination model, our observations were similar to the Kazanis study in that we found a greater contribution of local OPCs to oligodendrocyte regeneration but differed in that we saw a more significant influx of SVZ-derived OPCs by 28 dpl.

### 4.2. Temporal and Regional Determinants of Mobilizing Local OPCs and SVZ Cells

The difference between our findings and those from the cuprizone studies may be partly attributed to the temporal difference in the evolution of myelin damage and repair in the cuprizone and LPC models. The more robust remyelination, mediated by the progeny of NSCs in the cuprizone model, could be because the SVZ has had time to expand during the prolonged two to six weeks of demyelination stage, and that two to seven days of demyelination in the LPC lesion was too short for these responses to occur in the SVZ. The increased density of NSC-derived OPCs in the lesioned corpus callosum that we observed at 28 dpl is consistent with this, as is the observation that oligodendrocyte production from NSCs does not occur during the first four weeks [19]. Other studies that examined the response of more differentiated progeny of neural stem cells such as PSA-NCAM+ or Dcx+ neuroblasts in the SVZ have observed a more rapid response [16,18,32].

In the two cuprizone studies described above, the progeny of NSCs contribute significantly more to remyelination in the rostral corpus callosum, whereas local OPCs contribute more toward remyelination in the caudal corpus callosum. This could reflect the normal physiological dynamics of SVZ cells in rostral and caudal corpus callosum [38] and may reflect a greater abundance of oligodendrogliogenic progenitor cells in the caudal SVZ. In our experiments, LPC was injected into the rostral corpus callosum, and in most mice, demyelination spread laterally and caudally, so that the center of the demyelinated lesion typically occurred more than 500 μm caudal from the injection site. Thus, our observation that local OPCs generated oligodendrocytes before NSC-derived cells did reflect the location of the demyelinated lesion. In addition to the rostro-caudal difference, there is medio-lateral heterogeneity among NSCs, those with oligodendroglial fate potential being more abundant along the dorsal SVZ than in the dorsolateral angle, where neurogenic NSCs reside [39].

### 4.3. Plasticity of Neuronal and Oligodendrocyte Lineages in the SVZ

The SVZ constitutes one of the two neurogenic niches in the mammalian CNS where neural stem cells reside and continue to generate new neurons and glia in the adult. Cells isolated from the adult mouse SVZ undergo self-renewal and can be induced to generate astrocytes, neurons, and a few oligodendrocytes [14,39,40]. Under normal physiological conditions, oligodendrocytes are a minor fate among the cells of the SVZ [41]. The adult mouse SVZ consists of nestin+ GFAP+ neural stem cells, PSA-NCAM+ Dcx+ neuroblasts that migrate through the RMS to the olfactory bulb, and rapidly amplify type C cells, which express Distal-less homeobox 2 (Dlx2) and give rise to neuroblasts [15,42]. In the SVZ, committed oligodendrocyte lineage cells are sparse, and importantly, OPCs are distinct from GFAP+ neural stem cells, rapidly proliferating type C cells, and Dcx+ neuroblasts [43].

After acute demyelination in the corpus callosum, PSA-NCAM+/Dcx+ progeny of NSCs, likely to be neuroblasts, extensively proliferate and migrate out of the SVZ toward the demyelinated lesion [16,18]. In response to an inflammatory lesion, the number of oligodendrocyte lineage cells increase in the SVZ at the expense of neurons in the olfactory bulb, resulting in impaired olfactory memory, suggesting plasticity of neuronal and glial fates of SVZ stem cells [17]. However, because oligodendrocyte lineage cells, particularly OPCs, actively transcribe low levels of neuronally expressed genes such as Dcx, Dlx2, and glutamic acid decarboxylase 67 (Gad1) [2], it has been a challenge to study the fate of neuroblasts or type C cells [18,43]. Thus, it still remains unclear whether demyelination triggers reprogramming of normally neuronally committed NSCs or their progeny or amplifies oligodendrocyte-fated NSC cell clones.

### 4.4. Mechanisms that Could Affect Oligodendrogliogenesis from the SVZ

Local OPCs in the corpus callosum are capable of sensing a change in the degree of myelination or oligodendrocyte density and rapidly expand to repair the deficit [6,44]. Nestin+ NSCs, on the other hand, require two to three weeks to initiate the program to produce oligodendrocyte lineage cells, but given the necessary time, they can migrate and generate OPCs. How might a demyelinating lesion in the corpus callosum signal to the SVZ? Some proposed mechanisms include soluble factors such as netrin-1 [45] and epidermal growth factor [46] that promote emigration of cells out of the SVZ, and factors such as chordin [18], and Wnt7 [39] that promote oligodendroglial fate of NSCs. Besides these positive signals, negative regulators of oligodendrogliogenesis such as Gli1 [47], neurofibromin 1 [48], and Drosha [49] are beginning to be uncovered. How these mechanisms are coordinately regulated in response to demyelination remains to be elucidated.

## 5. Conclusions

In summary, we have shown that after acute demyelination in the corpus callosum, local OPCs rapidly expand and differentiate into remyelinating oligodendrocytes within the first two weeks. By contrast, NSCs in the SVZ begin their oligodendrogliogenic program with a temporal delay of two weeks, resulting in an increased population of SVZ-derived OPCs by four weeks after demyelination. The SVZ may be a limited source for repopulating OPCs, as it becomes depleted after sustained or repeated demyelination [20], which often occurs in chronic cases of multiple sclerosis (MS). Furthermore, the potential of SVZ-derived supply of OPCs in MS would be limited to lesions near the SVZ. Interestingly, NG2 cell density declines after remyelination of acutely demyelinated lesion in the spinal cord [3], while OPCs in the corpus callosum maintain their ability to self-renew even after prolonged cuprizone treatment for 12 weeks [20]. Further elucidation of the differences in the cellular properties of newly generated OPCs and those that have been residing in the white matter for an extended period of time could lead to new strategies to harness the ubiquitous population of local OPCs with enhanced ability for myelin repair.

## Figures and Tables

**Table 1.** Primary antibodies used.

Antibody	Host Species	Source	Dilution
Dcx	Rabbit	Cell Signaling Technology (Danvers, MA, USA)	1:300
NG2	Rabbit	EMD Millipore (Burlington, MA, USA)	1:500
Pdgfra	Goat	R&D Systems (Minneapolis, MN, USA)	1:1000
CC1 (Quaking 7)	Mouse	EMD Millipore (Burlington, MA, USA)	1:100
MBP, smi99 antibody	Mouse	Covance (Princeton, NJ, USA)	1:3000
Smi32	Mouse	Covance (Princeton, NJ, USA)	1:1000
GFP	Chick	Aves Labs (Tigard, OR, USA)	1:1000
Olig2	Mouse	EMD Millipore (Burlington, MA, USA)	1:1000
Olig2	Rabbit	Novus Biologicals (Littleton, CO, USA)	1:1000
PSA-NCAM, 12E3 antibody	Mouse	Dr. Tatsunori Seki (Tokyo Medical University, Tokyo, Japan)	1:1000
GFAP	Rabbit	DAKO-Agilent (Santa Clara, CA, USA)	1:2000

Dcx: Doublecortin; Pdgfra: Platelet-derived growth factor receptor α; MBP: Myelin basic protein; Smi32: Non-phosphorylated neurofilaments; GFP: Green fluorescent protein; Olig2: Oligodendrocyte transcription factor 2; PSA-NCAM: Polysialic acid-neural cell adhesion molecule; GFAP: Glial fibrillary acidic protein.
